# No evidence for systemic low‐grade inflammation in adult patients with early‐treated phenylketonuria: The INGRAPH study

**DOI:** 10.1002/jmd2.12366

**Published:** 2023-10-04

**Authors:** Chloé Giret, Yann Dos Santos, Hélène Blasco, Christophe Paget, Loïc Gonzalez, Nathalie Tressel, Maeva Dieu, Adrien Bigot, Valérie Gissot, Alexandra Audemard‐Verger, François Maillot

**Affiliations:** ^1^ Service de médecine interne CHRU de Tours Tours France; ^2^ Université de Tours Tours France; ^3^ UMR INSERM, “iBrain” 1253 Tours France; ^4^ Laboratoire de biochimie, CHRU de Tours Tours France; ^5^ Centre de référence des maladies héréditaires du métabolisme Tours France; ^6^ Centre d'étude des Pathologies Respiratoires (CEPR) UMR INSERM 1100 Tours France; ^7^ Centre d'investigation clinique, CHRU de Tours Tours France

**Keywords:** cardiovascular risk, C‐reactive‐protein, cytokines, inflammation, phenylketonuria

## Abstract

The question of an increased cardiovascular risk has been recently raised in adults with phenylketonuria (PKU). As low‐grade systemic inflammation increases cardiovascular risk, the INGRAPH study aimed to evaluate low‐grade inflammation in adult PKU patients compared to healthy controls and to determine the potential influence of Phe‐controlled diet on inflammation. Twenty early‐treated adult PKU patients, including a subgroup of 15 classical PKU patients, and 20 healthy volunteers were included. PKU patients and healthy subjects were matched on age, sex and body mass index class. Plasma concentrations of CRP, IFNg, IL1a, IL1b, IL2, IL6, IL10, and TNFα were measured in PKU patients and compared to controls. Plasma CRP was not different in the PKU group as compared to controls. No significant differences were observed between the two groups concerning plasma cytokines concentrations. Plasma CRP and cytokine profile were not different between “on diet” and “off diet” PKU patients. All these results were similar considering only the classical PKU subgroup. No differences were shown in plasma CRP and pro‐inflammatory cytokines between adult PKU patients and healthy controls. Further studies are needed, including more patients and extensive characterization of systemic low‐grade inflammation, as cardiovascular risk appears to be a new concern in adult PKU population.

**Synopsis**

The present study did not demonstrate any evidence for systemic low‐grade inflammation in adult early‐treated PKU patients. PKU diet and plasma phenylalanine seem to have no influence on inflammation markers.

## 1 INTRODUCTION

Phenylketonuria (PKU) is mostly due to mutations in the phenylanine hydroxylase (PAH) gene. When PAH activity is deficient, phenylalanine (Phe) is not converted to tyrosine and then accumulates in both blood and brain. Through several mechanisms, high concentrations of Phe are neurotoxic in untreated newborn and children, leading to development delay.^1^ Many countries have implemented a systematic newborn screening for PKU that allows early diagnosis and treatment using a Phe‐restricted diet as well as sapropterin in BH4‐responsive patients. Early treatment leads to normal development in case of good metabolic control.^2^ However, adult outcome is still considered as suboptimal, as adults with PKU may present some specific complications. Most concerns have been raised about neuropsychiatric complications along with behavior issues but there are new questions about systemic manifestations of PKU occurring at the adult age.^3^, ^4^, ^5^ As an example, an increase in cardiovascular risk in early‐treated adults with PKU has been suggested from the recent literature.

Burton et al.'s retrospective study, based upon collected data from insurance claims, has evaluated general health status in a population of 3691 PKU patients compared to non PKU controls and have shown that the highest adjusted prevalence ratios were for overweight and hypertension.^5^ Azabdaftari et al. showed that adult PKU patients have a significantly higher systolic and diastolic blood pressure, higher body mass index (BMI) and total cholesterol as compared to non PKU patients.^6^ Hermida‐Ameijeras et al. have also identified, in a study including 41 PKU patients versus healthy controls, a tendency to overweight and an increased aortic stiffness.^7^ Recently, Tanacli et al. showed that cardiac phenotype of PKU patients included some features of early‐stage cardiomyopathy.^8^

As far as cardiovascular risk is concerned, it is now well established that chronic inflammation is involved. In 1997, Haverkate et al. demonstrated in a prospective cohort that increased concentrations of C‐reactive protein (CRP) were predictive of coronary events in patients with stable or unstable angor.^9^ In 2000, Ridker et al. have shown that CRP and other markers of inflammation, such as serum amyloid A, interleukin‐6, and soluble intercellular adhesion molecule type 1, were predictors of cardiovascular disease in women.^10^ In a study involving 14 916 apparently healthy men, elevated levels of interleukin 6 (IL6) were associated with an increased risk for future myocardial infarction, which suggests a role for cytokine‐mediated inflammation in the early stages of atherogenesis.^11^ Roman et al. demonstrated that targeting inflammation with an anti‐inflammatory therapy can reduce recurrence in atherosclerotic cardiovascular disease events in patients with elevated plasma IL6, IL1, and CRP.^12^ In 2020, both CANTOS and COLCOT trials strongly suggested that using anti‐inflammatory drugs such as anti‐IL1 therapy and methotrexate could to reduce cardiovascular risk.^13^, ^14^

In a PKU murine model, it has been shown that low‐grade systematic inflammation, represented by slight increase in plasma CRP and pro‐inflammatory cytokines, exists and is reversible following a dietetic therapy using glycomacropeptide, through a probiotic effect of this protein which is naturally low in phenylalanine.^15^ In a small group of children and adults with PKU, Deon et al. were able to identify a pro inflammatory state with significantly increased plasma concentrations of both IL1B and IL6 compared to healthy controls.^16^ However, the patients included in this study were mainly adolescents who were late diagnosed. This work is, therefore, not truly attributable to the study of inflammation in the early‐treated adult PKU population. In a cohort of 20 adult patients with classical PKU, Mozrzymas et al. did not show any plasmatic increase of pro‐inflammatory cytokines including IL1B, IL6, and IL‐8.^17^ Such conflicting data in humans with PKU may be related to the small size of the evaluated cohorts, as well as the heterogeneity in patients included in studies published so far. Thus, existence of such potential low‐grade systematic inflammation, evaluated by CRP and plasmatic cytokine profile, has not been widely explored in adults with PKU. The aim of the present study (INflammation of low GRAde in PHenylketonuria [INGRAPH] study) was then to evaluate the low‐grade inflammatory profile in early‐treated adult PKU patients as compared to healthy controls, by measuring in both groups, plasma concentrations of CRP as well as pro‐inflammatory cytokine profile. The second aim of the study was to determine the potential influence of Phe‐controlled diet on such low‐grade inflammation.

## 2 PATIENTS AND METHODS

### 2.1 Patients

Patients with PKU were prospectively included from the adult outpatient clinic of our reference center for inherited metabolic diseases. The inclusion criteria were age ≥ 18 years old, confirmed diagnosis of PKU, early‐treated PKU following newborn screening, affiliation to the public national health insurance system. Both “on diet” and “off diet” PKU patients were included. Healthy volunteers were included by the clinical investigation center of our university hospital. Inclusion criteria were age ≥18 years old, no metabolic condition and affiliation to the public national health insurance system. For both PKU patients and healthy volunteers, noninclusion criteria were pregnancy and lactation, subject to legal protection measures, chronic or acute inflammatory disease, fever at time of inclusion, current anti‐inflammatory treatment, surgery within the previous month, diabetes, and hypertension.

### 2.2 Study design

The study was a monocentric, cross‐sectional pathophysiological study. Healthy volunteers were matched to PKU patients according to age, sex, and BMI class defined as normal for BMI < 30 kg/m^2^. All patients and healthy controls signed a written informed consent. French committee of persons protection of Paris Sud Est VI examined and approved the trial. The INGRAPH study identifier on ClinicalTrials.gov was NCT04879277.

### 2.3 Clinical and laboratory investigations

At inclusion visit, PKU patients and healthy controls were weighted and measured, allowing BMI calculation. The other data collected at inclusion were sex and age, blood pressure, and heart rate. Complete clinical examination was performed, and medical history was collected regarding cardiovascular disease, hypercholesterolemia, and history of comorbidities (alcohol/smoking) was gathered. Information about specific PKU therapy were also collected: Phe‐restricted diet (“on diet” or “off diet”), amino acids supplements intake, sapropterin therapy.

For healthy volunteers and PKU patients, fasting blood samples were collected using 1 EDTA tube of 6 mL and one tube of lithium heparin. Samples were sent to our biochemistry lab then centrifuged, aliquoted in four aliquots of 0.2 to 0.5 mL and stored at −80°C. Concentrations of plasma Phe was measured by Chromatography coupled to mass spectrometry. Plasma CRP was measured using an immunoturbidic method with the CRP4 kit (Roche Diagnostics, France) on Cobas C501 automate, CRP range being 0.3–350 mg/L using such method. Plasma cytokines were quantified in both groups using Human ProcartaPlex Mix&Match 7‐plex (Thermofisher, Waltham, USA), on a Luminex 100/200 (BioRad, Hercules, CA, USA). Plasma concentrations of IL2, IL10, Interferon gamma (INFg), IL1 alpha (IL1a), IL6, IL1 beta (IL1b), and TNF alpha were measured. The kit was used according to the supplier's recommendations.

### 2.4 Statistical analysis

Data are expressed as mean ± SD except indicated otherwise. Criteria judgment being multiparametric, calculation of population needed was not appropriate. As there are few data in literature, a number of subjects of 20 per group has been suggested. Statistical analysis was performed using GraphPad Prism software (version 8.2.1). All statistical tests were conducted in a two‐sided manner and the significance level set at *p* < 0.05. Analyses over BMI categories were performed using Kruskal–Wallis test. Welch *t*‐test was used for anthropometric data and PKU control comparison. Welch's ANOVA was used for control versus “on diet” versus “off diet” comparison. T3 Dunett's test was used for multiple comparison. Plasma CRP comparisons between groups were performed using Mann–Whitney test. As many cytokine concentrations were without any range of detection, we used fluorescent level over concentrations to compare both groups, to increase the statistical power of our study.^18^, ^19^ Fluorescence was normalized using a log10 transformation. First step on analysis was to compare normalized fluorescence (arbitrary unit) of inflammatory parameters between PKU patients and healthy patients. The second step was to compare fluorescence between BMI categories. Last analysis was to compare fluorescence according between “on diet” and “off diet” in a subgroup of classic PKU patients.

## 3 RESULTS

Twenty adult PKU patients and 20 matched healthy volunteers were included in the study. Among PKU patients, 15 had classical PKU, 3 patients had mild hyperphenylalaninemia, and 2 patients had mild PKU. Clinical and biological characteristics of both groups are summarized in Table 1. There was no statistical difference in clinical parameters between PKU and control groups. In the PKU group, 9 (45%) patients were “on diet” at the time of inclusion (7 in the classical PKU group). All “on diet” patients were consuming amino acids supplements. No patient was treated with sapropterin.

**TABLE 1:** Clinical characteristics of adult PKU patients and healthy controls, and comparison between groups.

	Adult PKU patients (*n* = 20)		Healthy controls (*n* = 20)		*p*
	Mean (±SD)	Median (1st Quartile–3rd Quartile)	Mean (±SD)	Median (1st Quartile–3rd Quartile)	
Age (y)	36.3 ± 8.5	36.5 (32.8–41.3)	36.6 ± 8.0	37 (32.8–41.8)	NS
Weight (kg)	77.2 ± 20.4	72.9 (63.15–95.8)	77.7 ± 20.5	73.4 (62.3–91.3)	NS
Height (cm)	164 ± 8.4	162.5 (157.8–171.4)	168.2 ± 7.1	166.8 (163.8–174.5)	NS
BMI (kg/m^2^)	28.4 ± 6.7	27.6 (23.5–31.8)	26.8 ± 6.8	25.3 (21.1–31.7)	NS
Sex	15 F–5 M		15 F–5 M		
“On diet”	9 (45%)				

*Note*: “On diet” means patients treated with a phenylalanine‐restricted diet.

In PKU patients, plasma Phe was 1179 ± 550 μmol/L. In the “on diet” PKU group (*n* = 9) and “off diet” PKU group (*n* = 11), plasma Phe concentrations were 1034 ± 420 and 1345 ± 658 μmol/L respectively. This result reflects poor metabolic control in the study population as none of the patients had Phe levels below the recommended target of the European guidelines. Plasma Phe concentrations were not significantly different between these two groups of PKU patients (*p* = 0.25).

**FIGURE 1:**
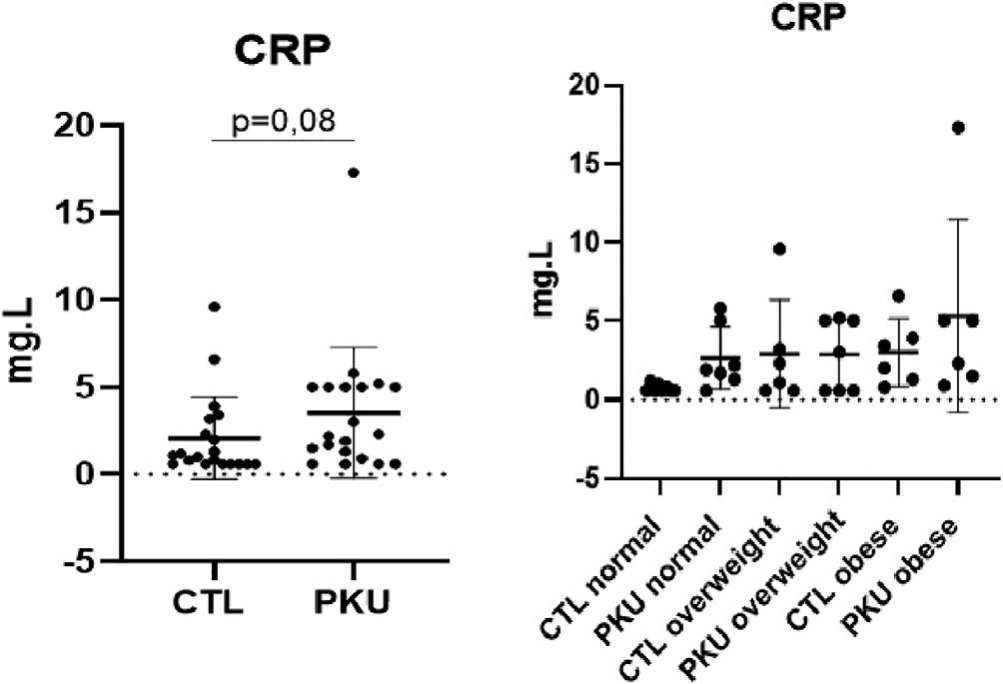
Comparison of plasma C‐reactive protein between adult phenylketonuria patients and controls (left), and according to BMI class (right). BMI classes = normal <25 kg/m^2^, overweight 25–30 kg/m^2^, obesity >30 kg/m^2^). No statistical differences between groups. CTL, controls.

Plasma CRP (Figure 1) was not statistically different in the PKU group as compared to healthy controls (3.5 ± 3.8 mg/L vs. 2.1 ± 2.4 mg/L, *p* = 0.08). There is a potential outlier in the PKU group, probably attributable to morbid obesity (BMI of this patient = 40.7 kg/m^2^). However, we have another patient with a BMI 40.9 kg/m^2^ with a CRP of 5.8 mg/L. Both patients had similar hyperphenylalaninemia (1681 and 1800 μmol/L respectively). To this regards, we decided to not exclude this potential outlier. Comparison of CRP between “on diet” (2 ± 1.8 mg/L) and “off diet” (4.7 ± 4.5 mg/L) PKU patients also showed no difference (*p* = 0.07).

**FIGURE 2:**
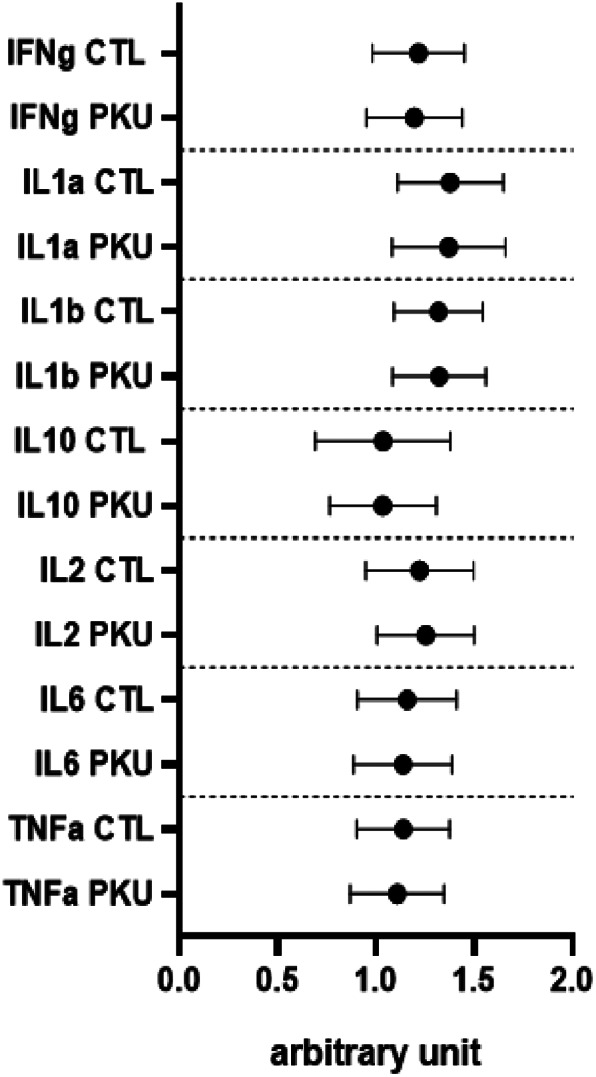
Comparisons of plasma cytokines between adult phenylketonuria (PKU) patients and healthy controls (CTL). No statistical differences have been observed between PKU patients and controls.

Regarding cytokines, plasma concentrations of IFNg (*p* = 0.7881), IL1a (*p* = 0.9374), IL1b (*p* = 0.9484), IL2 (*p* = 0.6985), IL6 (*p* = 07950), TNFα (*p* = 0.6791) were not statistically different between PKU patients and healthy controls (Figure 2). Plasma IL10, which is considered as an anti‐inflammatory cytokine, was not different between groups (*p* = 0.9953). Comparison between groups according to BMI showed that plasma cytokines concentrations did not differ significantly (Figures S1 and S2). Plasma cytokine profile was not different between “on diet” and “off diet” PKU patients.

Comparisons of biological data have also been performed in the 15 classical PKU patients versus 15 matched healthy controls, Plasma CRP was 5.5 ± 5.0 mg/L in classical PKU patients versus 2.2 ± 2.0 mg/L in controls (*p* = 0.08). Plasma cytokine profile comparisons between these two groups showed no statistical differences (see supplementary data).

## 4 DISCUSSION

The aim of the present study was to determine whether systemic low‐grade inflammation was detectable in adult early‐treated PKU patients. In the patients included in the INGRAPH study, plasma CRP was slightly increased in PKU patients versus controls, which could be indicative of increased cardiovascular risk, as Ross et al. suggested in 1999,^20^ but the difference observed did not reach statistical significance. Looking at the homogenous subgroup of patients with classical PKU which represents the most severe form of the disease, we did not find either any evidence for low‐grade systemic inflammation versus controls. These results indicate that the present study did not demonstrate any evidence for low‐grade inflammation in adult early‐treated PKU patients. Such result may be due to the lack of power of our study which has included a limited number of patients and controls. Therefore, our hypothesis has to be tested in a larger group of PKU patients.

In our comparisons between adults with PKU versus healthy controls, a confounding factor may be the impact of overweight and obesity as these conditions can be associated with systemic low‐grade inflammation^21^ that resolve following weight loss.^22^ Once more, we did not demonstrate any differences in plasma CRP concentrations according to BMI subclasses, probably because of the small sample of patients included. Finally, the sensitivity of plasma CRP can be questioned in our study, and some other markers of systemic inflammation may be of interest. As such in our study, we did not include serum amyloid A, other interleukins, and soluble intercellular adhesion molecules.

In the INGRAPH study, we did not demonstrate any plasma cytokines profile differences between adult PKU patients and controls. Mozrzymas et al. have measured plasma IL6 and IL8 levels in a group of young “on diet” adults with PKU diagnosed through neonatal screening.^17^ These authors did not detect any significant variation of cytokines as compared to controls, which is in agreement with our results, although 55% of our patients were “off diet.” This may suggest that following or not a Phe‐restricted diet in adulthood may not be associated with pro‐inflammatory mediators such as cytokines. In our study, being “on diet” versus “off diet” did not display any difference in terms of plasma cytokine profile as compared to controls. These assumptions should be taken with caution. Indeed, there was no significant difference in phenylalanine levels between the two groups, reflecting poor metabolic control in the “on diet” group. Data from Mozrzymas et al.'s study and the INGRAPH study are in contradiction with other previous publications. Deon et al. reported a significant increase in plasma IL1b and IL6 in PKU patients in a small number of patients whose clinical characteristics were different as compared to our patients.^16^ Indeed, PKU patients recruited in Deon et al.'s study were mostly adolescents who have been late diagnosed. At this stage, we cannot conclude whether late diagnosis of PKU can have an impact on inflammatory markers or not. In Stroup et al.'s study, a significant increase in bone‐related inflammatory cytokines including IL1β, IL17, TNF alpha, IL12, IFNg, IL6, and IL10 was observed in 27 adult PKU patients consuming amino acids medical food versus 254 healthy controls.^23^ In our study, we did not demonstrate that amino acids supplements intake could be associated with an abnormal inflammatory cytokine profile. At this stage, these conflicting data do not allow to draw any definite conclusions toward the existence of a systemic low‐grade inflammation in PKU in adulthood. Same divergent data have also been observed in patients with homocystinuria due to cystathionine beta‐synthase deficiency.^24^, ^25^ In PKU, further longitudinal research using multivariate analysis or a metabolomic approach should include large cohorts of adult patients, as only studies with a small number of patients, including the INGRAPH study, have been published so far.

As the present study did not demonstrate any detectable systemic signs of low‐grade inflammation in early‐treated adult patients with PKU, it was therefore not relevant to study the relationships between metabolic disturbances of PKU and inflammation. However, in a previous work including a cohort of 151 adults with PKU, we have shown some disturbances in tryptophan metabolism and more specifically in the kynurenine pathway.^26^ Plasma concentrations of kynurenines were lower as compared to the general population at the exception of kynurenic acid which was elevated. Interestingly, increase in plasma kynurenines has been shown to be associated with inflammation^27^ and cardiovascular mortality except for kynurenic acid.^28^ It is, therefore, conceivable that the decreased plasma kynurenines that we have observed can have some anti‐inflammatory effects and, as an opposite, elevated kynurenic acid may have a pro‐inflammatory effect. Some specific studies have to be performed to test these hypotheses.

In summary, our study showed no differences in plasma CRP and pro‐inflammatory cytokines between adult with early‐treated PKU patients and healthy controls. Our results also suggested that plasma Phe and BMI were not associated with inflammatory and proinflammatory markers in adult patients with PKU. Further studies are needed, including large numbers of patients and more complete characterization of the putative low‐grade systemic inflammation, as cardiovascular risk appears to be a new concern in adult PKU population.

## FUNDING INFORMATION

The INGRAPH study was funded by grants from “Les Feux Follets” (National French association of patients with PKU) and ADERMI (Association pour le Développement de l'Enseignement et la Recherche en Médecine Interne).

## CONFLICT OF INTEREST STATEMENT

The authors declare no conflicts of interest.

## ETHICS STATEMENT

The present study has been conducted in accordnace with to the ethical standards on human experimentation of our institution and with the Helsinki declaration of 1975, revised in 2013. (comment : the French committee of persons protection is the ethic committee).

## Supporting information


**Figure S1.** Plasma cytokines profiles in sub‐groups: “on diet” versus “off diet” PKU patients versus controls. The *p*‐values for each comparison are given below: all *p*‐values for IFNg are equal to 0.99; IL1a control versus “off diet,” *p* = 0.98; IL1a control versus “on diet,” *p* = 0.81; IL1a “on diet” versus “off diet,” *p* = 0.78; IL1b control versus “off diet,” *p* = 0.99; IL1b control versus “on diet,” *p* = 0.99; IL1b “off diet” versus “on diet,” *p* = 0.98; all *p*‐values for IL10 are equal to 0.99; IL2 control versus “off diet,” *p* = 0.93; IL2 control versus “on diet,” *p ≥* 0.99; IL2 “off diet” versus “on diet,” *p* = 0.96; All *p*‐values for IL6 are equal to 0.99; TNFa control versus “off diet,” *p* = 0.99, TNFa control versus “on diet,” *p* = 0.9, TNFa “off diet” versus “on diet,” *p* = 0.99.
**Figure S2.** Comparison of plasma cytokines according to BMI class subgroups. The *p*‐values for each comparison are given below: all *p*‐values for IFNg comparisons are equal to >0.99 except for the PKU overweight versus PKU obese, *p* = 0.18; all *p*‐values for IL2 comparisons are equal to >0.99 except for PKU overweight versus PKU obese, *p* = 0.19 and for control normal versus PKU obese, *p* = 0.75; all *p*‐values for IL1a comparisons are equal to >0.99 except for the PKU overweight versus PKU obese, *p* = 0.77; All *p*‐values for IL6 comparisons are equal to >0.99 except for the PKU overweight versus PKU obese, *p* = 0.12 and for control overweight versus PKU overweight, *p* = 0.6, All *p*‐values for IL1b comparisons are equal to >0.99 except for the PKU overweight versus PKU obese, *p* = 0.1 and PKU obese versus control obese, *p* = 0.72; All *p*‐values for IL10 comparisons are equal to >0.99 except for the PKU overweight versus PKU obese, *p* = 0.45 and for control overweight versus PKU overweight, *p* = 0.7; All *p*‐values for TNFa comparisons are equal to >0.99 except for the PKU overweight versus PKU obese, *p* = 0.17 and for control overweight versus PKU overweight, *p* = 0.49.

## Data Availability

The data that support the findings of this study are available from the corresponding author upon reasonable request.
