# Supplementary material for: No evidence for systemic low‐grade inflammation in adult patients with early‐treated phenylketonuria: The INGRAPH study
Source: JIMD Rep. 2023 Oct 4;64(6):446–52. doi: 10.1002/jmd2.12366 (PMC10623104; doi:10.1002/jmd2.12366)
Supplement: Supplementary file 1 — Figure S1. Plasma cytokines profiles in sub‐groups: “on diet” versus “off diet” PKU patients versus controls. The p‐values for each comparison are given below: all p‐values for IFNg are equal to 0.99; IL1a control versus “off diet,” p = 0.98; IL1a control versus “on diet,” p = 0.81; IL1a “on diet” versus “off diet,” p = 0.78; IL1b control versus “off diet,” p = 0.99; IL1b control versus “on diet,” p = 0.99; IL1b “off diet” versus “on diet,” p = 0.98; all p‐values for IL10 are equal to 0.99; IL2 control versus “off diet,” p = 0.93; IL2 control versus “on diet,” p ≥ 0.99; IL2 “off diet” versus “on diet,” p = 0.96; All p‐values for IL6 are equal to 0.99; TNFa control versus “off diet,” p = 0.99, TNFa control versus “on diet,” p = 0.9, TNFa “off diet” versus “on diet,” p = 0.99. Figure S2. Comparison of plasma cytokines according to BMI class subgroups. The p‐values for each comparison are given below: all p‐values for IFNg comparisons are equal to >0.99 except for the PKU overweight versus PKU obese, p = 0.18; all p‐values for IL2 comparisons are equal to >0.99 except for PKU overweight versus PKU obese, p = 0.19 and for control normal versus PKU obese, p = 0.75; all p‐values for IL1a comparisons are equal to >0.99 except for the PKU overweight versus PKU obese, p = 0.77; All p‐values for IL6 comparisons are equal to >0.99 except for the PKU overweight versus PKU obese, p = 0.12 and for control overweight versus PKU overweight, p = 0.6, All p‐values for IL1b comparisons are equal to >0.99 except for the PKU overweight versus PKU obese, p = 0.1 and PKU obese versus control obese, p = 0.72; All p‐values for IL10 comparisons are equal to >0.99 except for the PKU overweight versus PKU obese, p = 0.45 and for control overweight versus PKU overweight, p = 0.7; All p‐values for TNFa comparisons are equal to >0.99 except for the PKU overweight versus PKU obese, p = 0.17 and for control overweight versus PKU overweight, p = 0.49. [file JMD2-64-446-s001.docx]

**
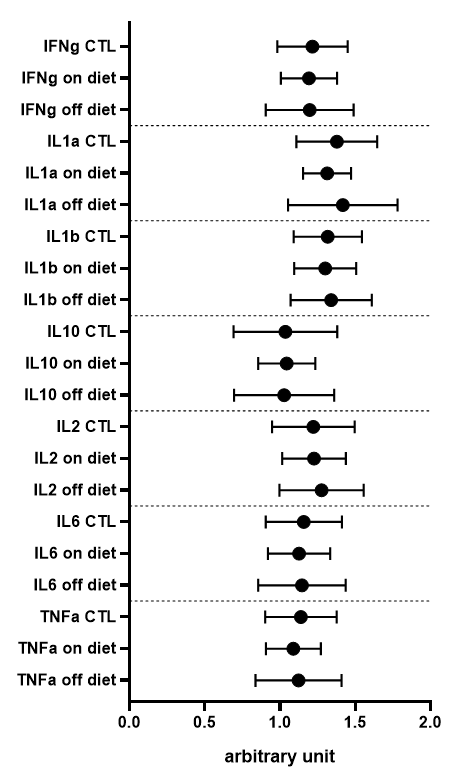
Supplementary data (including figures 3 and 4)**

**Figure 3.** Plasma cytokines profiles in sub-groups : “on diet” *vs* “off diet” PKU patients *vs* controls. The p-values for each comparison are given below : all p-values for IFNg are equal to 0,99 ; IL1a control vs “off diet”, p=0.98 ; IL1a control vs “on diet”, p=0,81 ; IL1a “on diet” vs “off diet”, p=0.78 ; IL1b control vs “off diet”, p=0.99 ; IL1b control vs “on diet”, p=0.99 ; IL1b “off diet” vs “on diet”, p=0.98 ; all p-values for IL10 are equal to 0.99 ; IL2 control vs “off diet”, p= 0.93 ; IL2 control vs “on diet”, p= >0.99 ; IL2 “off diet” vs “on diet”, p=0,96 ; All p-values for IL6 are equal to 0.99 ; TNFa control vs “off diet”, p=0.99, TNFa control vs “on diet”, p=0,9, TNFa “off diet” vs “on diet”, p=0,99.


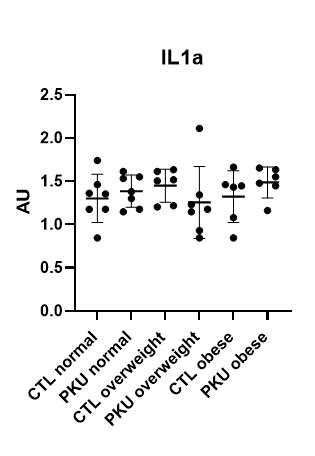

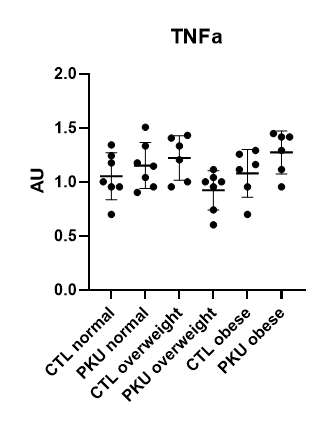

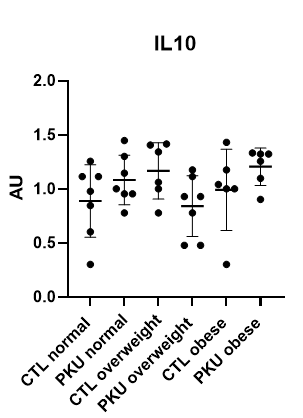

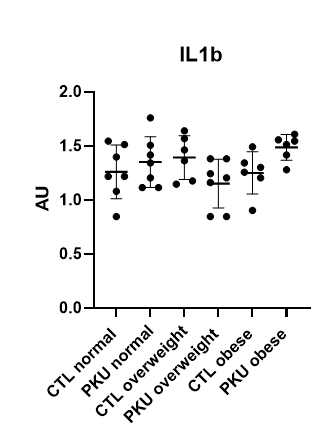

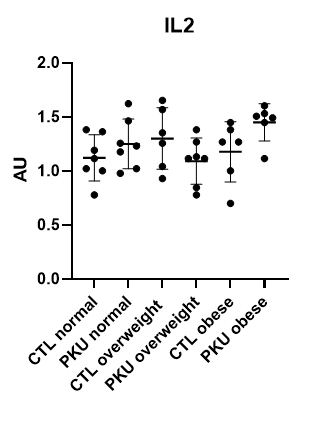

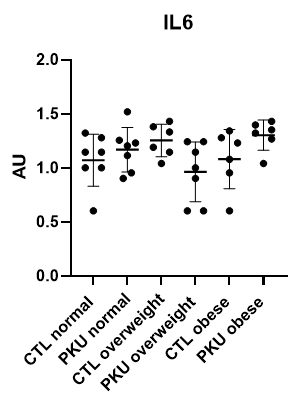

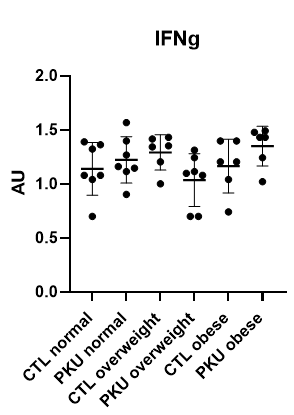


**Figure 4.** Comparison of plasma cytokines according to BMI class subgroups. The p-values for each comparison are given below: all p-values for IFNg comparisons are equal to >0.99 except for the PKU overweight vs PKU obese, p=0.18; all p-values for IL2 comparisons are equal to >0.99 except for PKU overweight vs PKU obese, p=0.19 and for control normal vs PKU obese, p=0.75 ; all p-values for IL1a comparisons are equal to >0.99 except for the PKU overweight vs PKU obese, p=0.77; All p-values for IL6 comparisons are equal to >0.99 except for the PKU overweight vs PKU obese, p=0.12 and for control overweight vs PKU overweight, p=0.6, All p-values for IL1b comparisons are equal to >0.99 except for the PKU overweight vs PKU obese, p=0.1 and PKU obese vs control obese, p=0.72 ; All p-values for IL10 comparisons are equal to >0.99 except for the PKU overweight vs PKU obese, p=0.45 and for control overweight vs PKU overweight, p=0.7 ; All p-values for TNFa comparisons are equal to >0.99 except for the PKU overweight vs PKU obese, p=0.17 and for control overweight vs PKU overweight, p=0.49.
